# Characterization of the key aroma compounds in three types of bagels by means of the sensomics approach

**DOI:** 10.1186/s13065-021-00743-4

**Published:** 2021-03-13

**Authors:** Ola Lasekan, Fatma Dabaj, Megala Muniandy, Nurul Hanisah Juhari, Adeseye Lasekan

**Affiliations:** 1 Department of Food Technology, University Putra Malaysia, UPM, 43400 Serdang, Malaysia; 2 Department of Food Service and Management, University Putra Malaysia, UPM, 43400 Serdang, Malaysia; 3 Food Science &Human Nutrition, School of Food & Agriculture, University of Maine, Orono, ME USA

**Keywords:** Bagel, Aroma-active compounds, Cold fermentation, Sensomics approach

## Abstract

**Background:**

To evaluate the impact of cold fermentation time on bagel rolls, the key aroma-active compounds in the volatile fractions obtained from three different bagel rolls through solvent assisted flavor evaporation (SAFE) were sequentially characterized by an aroma extract dilution analysis (AEDA), quantified by stable isotope dilution and analyzed by odor activity values (OAVs) respectively.

**Results:**

Findings revealed 40 aroma-active compounds with flavor dilution (FD) factor ranges of 2–1024. Of these, 22 compounds (FD ≥ 16) were quantified by stable isotope dilution assays (SIDA). Subsequent analysis of the 22 compounds by odor activity values (OAVs) revealed 14 compounds with OAVs ≥ 1 and the highest concentrations were obtained for 2,3-butanedione, 2-phenylethanol, 3-methylbutanal and acetoin respectively. Two recombination models of the bagels (i.e. 24 h and 48 h bagels) showed similarity to the corresponding bagels. Omission tests confirmed that 2,3-butanedione (buttery), acetoin (buttery), 2-acetyl-1-pyrroline (roasty), 5-methyl-2-furanmethanol (bread-like), (Z)-4-heptenal (biscuit-like) and 4-hydroxy-2,5-dimethyl-3(2H)-furanone, were the key aroma compounds. Additionally, acetic acid, butanoic acid, 2-phenylethanol (honey-like), 3-methylbutanoic acid, 2/3-methylbutanal, vanillin, 3-methylbutanol, methional were also important odorants of the bagel.

**Conclusion:**

Whilst the long, cold fermented bagels exhibited roasty, malty, buttery, baked potato-like, smoky and biscuit-like notes, the control bagels produced similar but less intense odor notes.

## Introduction

Bagels are one of the most widely consumed bread rolls in the United States. Recent statistics have shown that 204 million Americans consumed bagels in 2019 [1]. This figure is projected to increase to 210 million in 2023 [1]. Bagels have a very simple formulation similar to simple bread or roll formulas (i.e. flour, salt, yeast, and water). However, what differentiates bagel from the rest of the rolls are the flour quality and the long, cold fermentation of the dough used in bagel production. Traditional bagels are often produced with high protein (13–16%) spring wheat flour [2]. In addition, the long, cold fermentation step called retardation gives the traditional bagels a distinctive crust and flavor not found in the regular bread rolls. The quality of bread is normally defined by its volume, texture, color and flavor [3]. However, the aroma of bread is undoubtedly one of the most important qualities that influence its acceptance by consumers [4].

Bread flavor appreciation is one of the first evaluation signals encountered by consumers during bread consumption [5]. The flavor of bread is engendered by the interaction of a large number of compounds, which exhibit different olfactive characteristics, tactile oral and trigeminal sensations. Some of these compounds include; alcohols, aldehydes, esters, ketones, acids, hydrocarbons, pyrazines, pyrrolines, furans etc. [3, 4]. Over 300 volatile compounds have been reported in white bread [6]. In addition, the odour quality of bread depends on many factors like; type of flour, type of fermentation [7] and dough improvers [8] used during bread production. The production process and storage are also known to influence the flavor of bread [9].

Analysis of volatile compounds in a food matrix is quite complex and several extraction methods have been reported, ranging from solvent extraction [10], headspace solid phase micro-extraction (HS-SPME) [11], dynamic headspace extraction (DHE) [4], multiple headspace solid phase micro-extraction (MH-SPME) [12], solvent assisted flavor evaporation (SAFE) [13] and vacuum sublimation [14]. In the same vein, many identification techniques have been employed to provide aroma profiles for different types of breads. Some of the techniques involved the use of gas chromatography–mass spectrometry alone [15] or coupled with a comprehensive bi-dimensional gas chromatography–time of flight mass spectrometry (GC × GC–TOFMS) [16], electronic nose [17], artificial mouth [18]. And proton-transfer-reaction mass spectrometry (PTR-MS) [19], which only provides the chemical identities of breads.

Recently, the sensomics approach, which includes, gas chromatography–olfactometry (GC–O), sensory analysis, aroma extraction dilution analysis (AEDA), identification experiments, quantitation by stable isotope dilution assays (SIDAs), calculation of odour activity values (OAVs) and aroma recombination and omission tests to validate analytical data, has proven a useful method for characterizing the potent aroma constituents of food [20]. Sensomics is a multi-step analytical procedure used for identifying and quantifying key odorants in a food matrix as well as defining their sensory impact on the overall food aroma [21, 22]. Sensomics approaches help to identify potent aroma compounds as well as taste components in food [23]. Furthermore, the sensomic approach combines separation-based chromatographic methods with reconstitution and omission experiments to evaluate the role of specific compounds in the perceived aroma of a mixture addition. The implication of this that the sensomics approach is able to produce a flavor-cum taste signatures of food [24]. In addition, the sensomics approach has been applied in the characterization of aroma compounds in yeast dough dumpling [22] and the crust of soft pretzels [25].

Although there are many reported studies on the characteristic aroma profiles of different wheat breads, however, there has been no reported study on bagels. In addition, bagel processing is slightly different from that of regular bread. Therefore, elucidating the flavor chemistry of bagels could improve their quality control and processing of bagels. The objective of this study was to characterize the key aroma compounds in long, cold fermented bagels using the sensomics approach.

## Materials and methods

### Bagel production

**Fig. 1:**
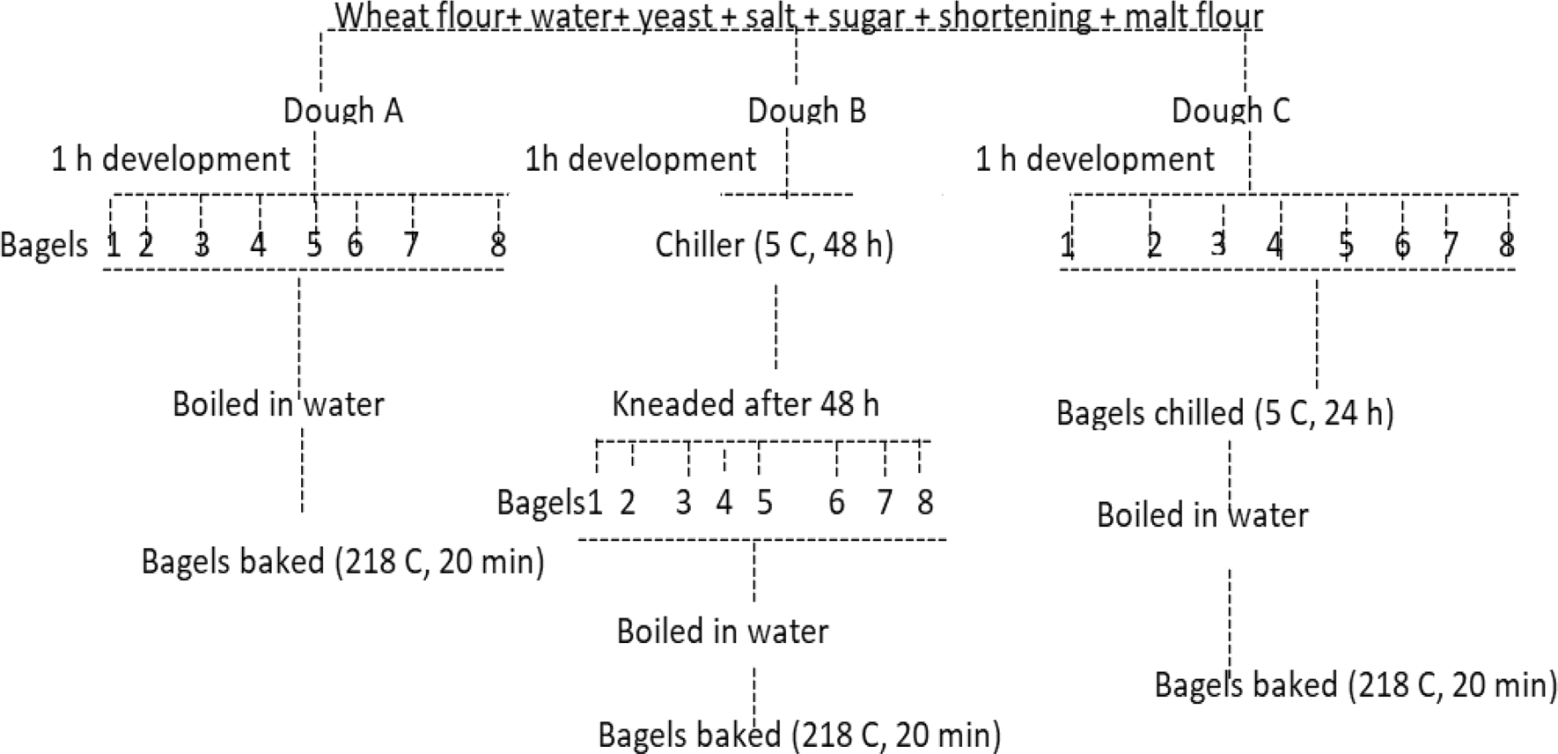
Flow diagram for the production of bagels

Bagel doughs were made by employing three processes differing in their cold fermentation conditions, and the time required for boiling the bagel dough in water (Fig. 1). The dough recipes contained high protein wheat flour 13% (enriched bakers patent flour from Pastry Product Sdn., Malaysia) (2000 g); cold water, 1100 g; instant dry yeast, 30 g; salt, 30 g; granulated sugar, 60 g, shortening; 60 g and malt flour (high diastatic malted barley flour 185 Lintner minimum) 60 g. The ingredients were made into dough by mixing it for 3 min in a mixer (Model VCM-44A-1, Stephan, Hameln, Germany). The dough was subsequently divided into 3 equal parts (dough A, B & C). Dough A (control) was kneaded for 10 min and allowed to develop for about 1 h. After 1 h, the dough was further kneaded a dozen times and divided into eight pieces. Each dough piece was rolled into a rope and the two ends were joined together to form a circle with a diameter of approximately 1–2 inches. The bagels were dropped into a large boiling water pot and allowed to boil for 2 min with constant turning. The boiled bagels were baked in a pre-heated oven at 218 °C for 20 min. Dough B was kneaded (10 min) as in dough A and allowed to develop for 1 h. After the kneading operation, the dough mass was returned into a large bowl, covered tightly and kept in a chiller (5 °C) for 48 h. After, 48 h of cold fermentation, the dough was brought out and kneaded for about 3 min and it was divided into eight pieces and made into eight bagels as described above. The bagels were boiled in water for 2 min and later baked in a pre-heated oven at 218 °C for 20 min. Dough C was kneaded as in dough A and allowed to develop for 1 h. After the kneading, the dough was divided into eight bagels. The pre-formed bagels were kept in the chiller (5 °C) for 24 h. After 24 h, the bagels were subjected to the same boiling and baking protocols as described above.

### Chemicals

Pure chemical standards with purity ranging between 97 and 99% were used. The chemical standards included; acetic acid, butanoic acid, benzyl alcohol, 2,3-butanedione (diacetyl), heptanoic acid, 3-hydroxy-2-butanone (acetoin), and octanoic acid which were purchased from Merck (Darmstadt, Germany). Ethyl nonanoate, ethyl octanoate, 4-hydroxy-2,5-dimethyl-3(2H)-furanone (HDMF), (*E*)-2-nonenal, 2-phenyl ethanol, phenyl acetaldehyde, 3-methylbutanoic acid, 3-methylbutanol, vanillin, 1-butanol, propionic acid, hexanoic acid, benzaldehyde, (*E,E*)-2,4-decadienal, furfural, 2-methylbutanal, 2,3-hexanedione, 2-heptanone, 5-methyl-2-furanmethanol, decanol, 4,5-epoxy-(*E*)-2-decanal, 1-octen-3-one, methional, 2-acetyl-1-pyrolline, sodium citrate dehydrate, and citric acid were from Aldrich (Steinheim, Germany) and 4-vinyl-2-methoxyphenol was purchased from Lancaster (Eastgate, Morecombe, UK). Ethanol (40% v/v) was of food grade. The following labelled compounds (Table 1) were synthesized according to the literature cited; [^2^H_2_]-butanoic acid [26]; 2-[^2^H_2_]-phenylethanol [27]; 3-[^2^H_2_]-methylbutanol [27]; [^2^H_2_]-ethyl octanoate [27]; [^13^C_2_]-acetic acid [28]; 3-[^2^H_2_]-methylbutanal [29]; [^2^H_2_]-2-acetyl-1-pyrroline [30]; [^13^C_4_]-2,3-butanedione [31]; [^2^H_2_]-3-methylbutanoic acid [32]; [^13^C_6_]-2-methoxy vinyl phenol [33]; [^2^H]-vanillin [33]; [^2^H_2_]-phenyl acetaldehyde [34]; [^2^H_2_]-heptenal [35] and 4-hydroxy-2[^13^H_2_]-methyl-5-methyl-3(2H)-furanone [36]. Citrate buffer (0.1 M, pH 6.0) was prepared as follow: sodium citrate dehydrate (12.044 g, 0.04 M) was added to 800 mL of distilled water in a liter round bottom flask with constant stirring. Subsequently, citric acid (11.341 g, 0.06 M) was added to the solution and the solution was adjusted to a pH 6.0 with 0.1 N HCl.

**Table 1:** Selected ions, and calibration factors used for the quantification of aroma compounds in three bagels by stable isotope dilution assays

No.	Compounds^a^	Selected ions (m/z)	Internal standards	Selected ions (m/z)	Calibration factor^b^
1	Phenylethanol	105	2-[^2^H] phenylethanol	107	1.02
2	Butanoic acid	89	[^2^H] butanoic acid	91	0.89
3	3-Methylbutanol	71	3[^2^H] methylbutanol	73	0.87
4	Acetic acid	61	[^13^C_2_] acetic acid	63	1.00
5	Ethyl octanoate	173	[^2^H] ethyl octanoate	176	1.00
6	4-Hydroxy-2,5-dimethyl-3(2H)-furanone	129	4-Hydroxy-2[^13^C] methyl-5-methyl-3(2H)-furanone	131	1.00
7	2,3-Butanedione	87	[^13^C_4_]-2,3-butanedione	91	0.90
8	3-Methylbutanal	87	[^2^H]-3-methylbutanal	89	1.00
9	3-Methylbutanoic acid	60	[^2^H]-3-methylbutanoic acid	62	1.00
10	Methional	105	[^2^H]-methional	108	1.00
11	2-Acetyl-1-pyrroline	112	[^2^H]-2-acetyl-1-pyrroline	114	1.00
12	Phenylacetaldehyde	121	[^2^H]-2-phenylacetaldehde	123	0.85
13	2-Methoxy-4-vinylphenol	150	[^13^C_6_]-2-methoxy-4-vinylphenol	156	0.85
14	Vanillin	137	[^2^H]-vanillin	139	0.98
15	(*Z*)-4-Heptenal	95	[^2^H]-(Z)-4-heptenal	97	0.98

Calibration factors^b^ and compounds^a^ were determined as earlier reported by Guth and Grosch [32] and Lasekan et al. [41] respectively

### Isolation of volatile constituents

Freshly baked bagels were sliced into pieces, frozen in liquid nitrogen and pulverized in a Waring blender. The pulverized bagel (300 g) was extracted with dichloromethane (700 mL) at room temperature (29 °C) for 2 h and the obtained extract was subjected to solvent-assisted flavor extraction (SAFE) distillation [13] at 40 °C. To separate the acidic volatiles from the neutral-basic fraction, the extract was treated four times with 50 mL of 0.5 mol L^−1^ aqueous sodium bicarbonate. The combined aqueous solutions were adjusted to pH 2 with HCl (2 mol L^−1^) and extracted with 50 mL of dichloromethane (4×) to obtain the acidic fractions. Subsequently, the solutions (i.e. the acidic or the neutral-basic) were concentrated to 2 mL at 40 °C using a small size Vigreux column [37]. The concentrated extract was further concentrated to 200 μL [38]. All analyses were repeated in triplicate.

### Analysis of volatile constituents

The GC–MS was performed by means of a gas chromatograph type QP-5050A (Shimadzu, Kyoto, Japan) using the following capillary columns: DB-5 (30 m × 0.25 mm I.D; 0.25 µm film thickness; Scientific Instrument Services, Inc., Ringoes, NJ); DB-FFAP (30 m, 0.32 mm I.D; 0.25 µm film thickness, Scientific Instrument Services, Inc., Ringoes, NJ). The extracts (2 µL) were applied by the on-column injection technique at 230 °C. The temperature of the oven was raised at 40 °C min^−1^ to 50 °C, held for 2 min isothermally and then raised at 4 °C min^−1^ to 250 °C. The flow rate of the carrier helium was 2.0 mL min^−1^. The retention indices (RI) of the compounds were calculated as described previously [37].

Mass spectra were recorded in the electron impact positive mode (EI) over a scan ranges of m/z 40–270 (scan frequency 5.8 Hz) applying electron energy of 70 eV. Total run time was 45 min. Source and transfer line temperatures were 200 and 240° C respectively. Mass spectra were evaluated by using the Xcalibur software (Thermos Scientific, Dreieich, Germany).

### GC–olfactometry

To further identify the aroma constituents in the bagel extracts, an olfactory detection port ODP-3 (Gerstel, Mulheim, Germany) connected to a Trace Ultra 1300 gas chromatograph (Thermos Scientific, Waltham, MA, USA) was used. The GC–O system was fitted with a DB-FFAP column (30 m × 0.32 mm i.d; film thickness, 0.25 µm, Scientific Instrument Services, Inc., Ringoes, NJ). The GC–O conditions are the same as reported in “Analysis of volatile constituents” section. The effluent was split 1:1. Sniffing was conducted as described previously [39]. Three experienced panelists (two females and a male) with strong gustative and olfactory responses in earlier sessions were used for the sniffing test. The sniffing analysis was divided into three sessions of 20 min and each assessor participated in the exercise. All analyses were repeated in triplicate by each assessor.

### Aroma extracts dilution analysis (AEDA)

The flavor dilution (FD) factors of the aroma compounds were determined by GC–O as reported by Lasekan and Yap [39]. The original extracts (200 µL) containing the neutral/basic as well as the acidic volatile compounds obtained from the crumbs (300 g) were diluted in a stepwise fashion by the addition of dichloromethane as described earlier [39]. Three panelists evaluated all dilutions in triplicate. Only the aroma compounds detected by more than two panelists were recorded. The flavor dilution factors obtained by AEDA [40] were plotted against the retention index values of the corresponding aroma compound (FD chromatogram).

### Aroma compound quantification by stable isotope dilution assays (ACQSIDA)

Labelled standards (20–50 µg) previously dissolved in dichloromethane (5 mL) were added to each crumb (100 g). The obtained extract was subjected to SAFE distillation as described earlier in “Isolation of volatile constituents”. Aliquots (0.5 µL) of the concentrates were analyzed by means of two dimensional GC–MS as described previously [41]. Calibration factor for each compound was determined by analyzing mixtures of defined quantity of the labelled compounds in five different mass ratios (1:5, 1:3, 1:1, 3:1, and 5:1) using the GC–MS. The obtained response factors from the peak area and the amounts of labelled compound are shown in Table 1. The concentration of compounds quantified by the selected stable isotopologues is reported in Table 3.

### Orthonasal aroma analysis of bagel

One hour after baking, the bagels (approximately 8 g with similar crust covering) were placed inside glass beakers (height 7 cm, volume 45 mL) with three random digitals and were orthonasally evaluated by panel members at room temperature (29 ± 2 °C). In addition, samples were rotated among panelists to prevent carry-over effects. The panel consisted of 10 members, aged between 24 and 35 years and were made up of seven women and three men. These panelists have participated in a weekly sensory training session for at least a year to be able to recognize and describe different aroma qualities. The sensory analyses were conducted in a sensory room following the International Standard (ISO 8589, 2007) [42] protocols with individual booths equipped with uniform and glare free white light (D65). Descriptors used were determined in preliminary sensory experiments as described by Steinhaus et al. [43]. The panelists started with seven descriptors and when all panelists were able to achieve complete agreement on a descriptor such a descriptor was chosen. Each descriptor used was defined on the basis of the odour of the selected aqueous solution of reference compounds. The reference compounds used as stimuli were; 10 μg L^−1^ of 2-acetyl-1-pyrroline (roasty); 100 μg L^−1^ of 3-methylbutanal (malty); 70 μg L^−1^ of 2,3-butanedione (buttery); 50 μg L^−1^ of (*Z*)-4-heptenal (biscuit-like); 10 μg L^−1^of 4-vinyl-2-methoxyphenol (smoky); 100 μg L^−1^ of methional (baked potato-like). During evaluation, the panelists had 5 min to rest after each set of samples was tested. All samples were repeated in triplicate. The intensities of the attributes were rated on a 7-point linear scale (i.e. 0, 0.5, 1.0, 1.5, 2.0, 2.5, 3.0) from 0 (not perceivable) to 3 (strongly perceivable) in steps of 0.5 by the panelists. The sensory data were analyzed by one-way analysis of variance (ANOVA) using SPSS 20.0 (SPSS Inc., Chicago, IL., USA). ANOVA with Duncan’s multiple comparison tests were performed to determine whether there were differences among individual samples. The differences were considered to be significant at p < 0.05 (Table 4). In addition, the ethical standards as instituted by the institutional and/or national research committee according to the 1964 Helsinki declaration and its later amendments or comparable ethical Standards on studies involving human subjects were adhered to. The study protocol and consent procedure received ethical approval from the Institutional Review Board (IRB) of the University Putra Malaysia. Informed consent was obtained from all individual participants included in the study.

### Aroma model recombinant of the 24 and 48 h bagels

Reference standards of key aroma compounds (Table 5) were prepared in ethanolic solution [44]. The combined ethanolic stock solutions of the 17 aroma compounds made up of 15 compounds with OAVs > 1 and two compounds (i.e. acetic acid and acetoin) with significantly high concentrations (Table 5) (500 μL) was added to citrate buffer (30 mL; pH 5.6; 0.1 mol L^−1^) and free corn starch (30 g) respectively in a closed Teflon cup. The Teflon cup was stirred continuously for 15 min at room temperature 29 °C. The aroma model was evaluated orthonasally in comparison with the 24 and 48 h bagels as described above (“Orthonasal aroma analysis of bagel” section).

### Omission experiments

A triangle test was performed to determine the significance of one odorant on the aroma recombination models (24 h and 48 h) reported in Table 5. For each of the models a glass of the mixture (20 mL) was prepared by omitting one or a group of selected odorants from the complete recombination model (Table 6). This mixture and two other glasses containing the complete recombination models were presented to the sensory panel in a triangle test [45]. The results of the Triangle tests were analyzed by comparing the total number of correct responses with the minimum number of responses required for statistical significance (ISO, 4120, 2004) [46]. Panel performance was obtained by applying analysis of variance (ANOVA) to the sensory profile data. The data were analyzed using SAS Statistical software (SAS Institute, Inc. 1996). The significance α was calculated according to the method of Callejo et al. [45]

## Results and discussion

### Identification of aroma-active compounds in control bagels

**Fig. 2:**
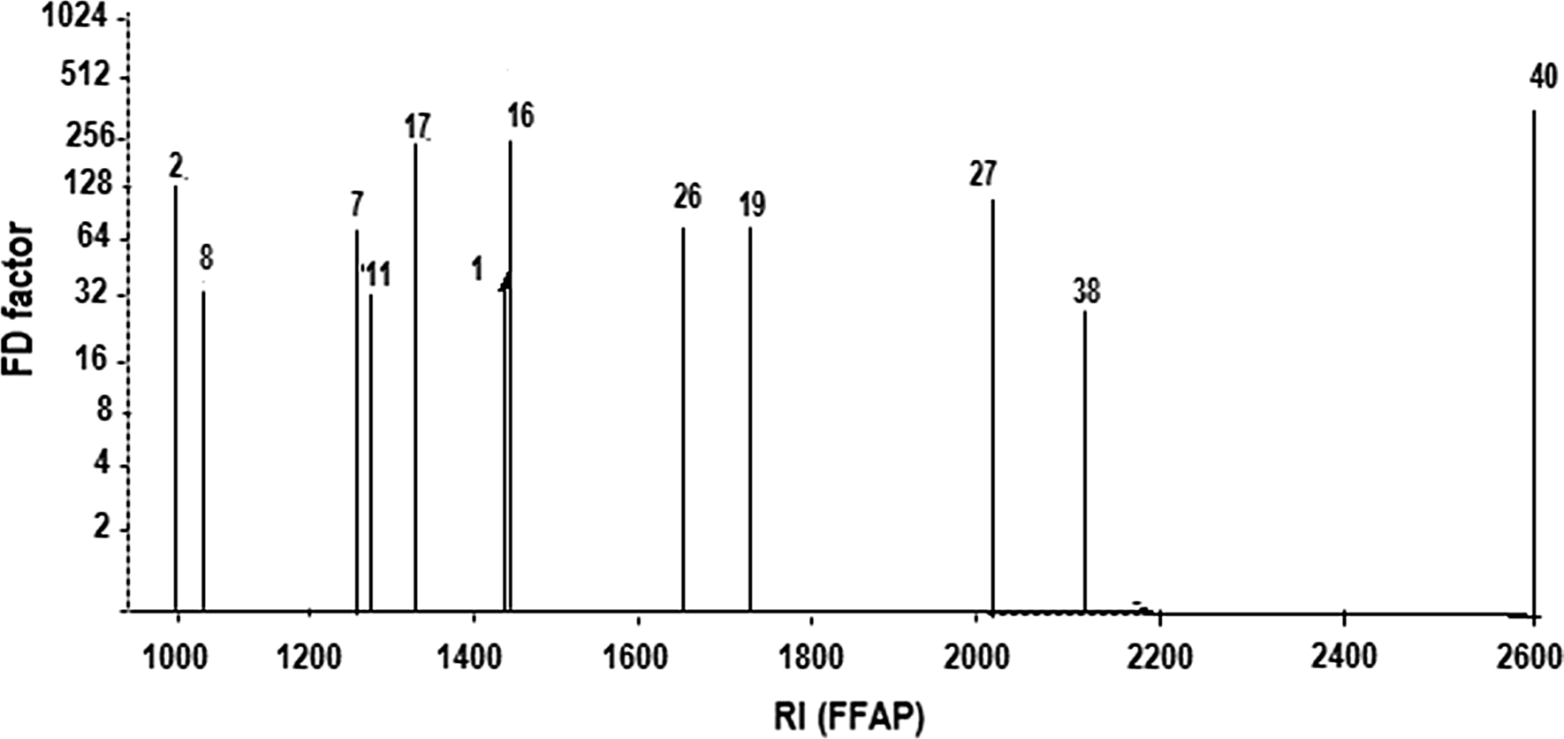
Flavour dilution chromatogram obtained by the application of AEDA on a distillate of unfermented bagel (control). Compounds with an FD factor ≥ 32 are displayed. Numbering is identical with that in Table 2

A combined total of 40 aroma compounds were identified in the three differently processed bagels (i.e. control; 24 h cold fermented bagels: and bagels produced from 48 h cold fermented dough mass). Among these compounds, 10 aldehydes, 9 alcohols, 7 acids, 6 ketones, 5 heterocyclic compounds and 3 esters were positively identified (Table 2). To reveal the differences between the flavors of the bagels, the volatile fractions of their crumbs were subjected to AEDA. In the control bagels, 40 aroma compounds were detected in the FD factor range of 2 to 256 respectively (Table 2). Furthermore, the results revealed 2-acetyl-1-pyrroline (roasty), methional (baked potato-like), vanillin (vanilla-like), 2,3-butanedione (buttery) and 4-hydroxy-2,5-dimethyl-3(2H)-furanone (HDMF) as compounds with the highest FD values in the control bagel. These aroma-active compounds exhibited high FD factors (128–256) (Fig. 1). Other important aroma compounds in the control bagels were butanoic acid (sweaty), acetoin (buttery), benzaldehyde (almond-like), furfural (bread-like), 2/3-methyl butanoic acid (sweaty), acetyl pyrazine (toasty), phenyl acetaldehyde (honey-like), 2-phenylethanol (honey-like), octanoic acid (fatty, soapy), 4-vinyl-2-methoxyphenol (smoky), acetic acid (sour), 3-methyl butanol (malty), and 2-methylpyrazine (nutty) all of which exhibited FD factors ranging from 16 to 32 (Fig. 2).

**Table 2:** Aroma compounds identified in cold fermented and control bagels

No.	Compound^a^	Retention index on DB-5	Retention index on FFAP	Odour description^b^	Fractions	FD CB	FD BF_24_	FD BF_48_	Identification method
1	Acetic acid	605	1443	Sweaty	A	32	64	64	MS/RI/O/ST
2	2,3-Butanedione (diacetyl)	606	993	Buttery	NB	128	512	1024	MS/RI/O/ST
3	1-Butanol	636	1179	Sweaty/buttery	NB	4	4	8	MS/RI/O/ST
4	2/3-Methylbutanal	647	936	Malty	NB	8	16	16	MS/RI/O/ST
5	Propionic acid	668	1540	Sweaty/pungent	A	8	8	8	MS/RI/O/ST
6	Butanoic acid	718	1619	Sweaty	A	16	64	64	MS/RI/O/ST
7	Acetoin	720	1275	Buttery	NB	16	128	128	MS/RI/O/ST
8	3-Methyl butanol	769	1067	Malty	NB	32	32	32	MS/RI/O/ST
9	2,3-Hexanedione	792	ND	ND	NB	ND	ND	ND	MS/RI/ST
10	Furfural	826	1457	Bread-like	NB	16	16	16	MS/RI/O/ST
11	2-Methyl pyrazine	827	1298	Nutty, roasty	NB	32	32	32	MS/RI/O/ST
12	2/3-Methylbutanoic acid	831	1661	Sweaty	A	16	64	128	MS/RI/O/ST
13	Isoamyl acetate	878	1124	Fruity	NB	8	16	16	MS/RI/O/ST
14	2-Heptanone	889	1182	ND	NB	ND	ND	ND	MS/RI/ST
15	Heptanol	896	1174	Citrusy	NB	2	4	8	MS/RI/O/ST
16	Methional	919	1449	Baked potato	NB	256	256	256	MS/RI/O/ST
17	2-Acetyl-1-pyrroline	922	1331	Roasty	NB	256	256	256	MS/RI/O/ST
18	Benzaldehyde	936	1196	Almond-like	NB	16	32	32	MS/RI/O/ST
19	5-Methyl-2-furanmethanol	953	1723	Bread like	NB	8	8	8	MS/RI/O/ST
20	(*Z*)-4-Heptenal	960	1287	Biscuit-like	NB	8	16	32	MS/RI/O/ST
21	Hexanoic acid	961	1842	Sweaty	A	4	8	8	MS/RI/O/ST
22	1-Heptanol	970	ND	ND	NB	ND	ND	ND	MS/RI/ST
23	1-Octen-3-one	971	1297	Mushroom-like	NB	2	4	4	MS/RI/O/ST
24	2,3,5-Trimethylpyrazine	985	1395	Broth-like	NB	2	4	4	MS/RI/O/ST
25	2-Pentyl furan	992	ND	Fruity, sweet	NB	4	4	8	MS/RI/O/ST
26	Acetyl pyrazine	1020	1662	Toast	NB	16	16	32	MS/RI/O/ST
27	4-Hydroxy-2,5-dimethyl-3(2H)-furanone	1022	2038	Sweet/caramel	A	128	512	1024	MS/RI/O/ST
28	Benzyl alcohol	1039	1866	Sweet/flowery	NB	8	16	16	MS/RI/O/ST
29	Phenyl acetaldehyde	1042	1653	Honey, rose	NB	16	16	16	MS/RI/O/ST
30	Heptanoic acid	1077	1949	Rancid	A	2	8	8	MS/RI/O/ST
31	2-Phenyl ethanol	1136	1911	Honey-like	NB	16	16	16	MS/RI/O/ST
32	(*E*)-2-Nonenal	1164	1568	Fatty, green	NB	8	8	8	MS/RI/O/ST
33	Octanoic acid	1182	2047	Fatty, soapy	A	16	32	32	MS/RI/O/ST
34	Ethyl octanoate	1194	1428	Fruity, fatty	NB	8	8	8	MS/RI/O/ST
35	Decanol	1269	ND	Fatty	NB	4	8	8	MS/RI/O/ST
36	Ethyl nonanoate	1296	ND	Fruity, tropical	NB	4	4	4	MS/RI/O/ST
37	(*E,E*)-2,4-Decadienal	1313	1684	Fatty	NB	2	4	8	MS/RI/O/ST
38	4-Vinyl-2-methoxyphenol	1317	2174	Smoky	A	16	32	32	MS/RI/O/ST
39	4,5-Epoxy-(E)-2-decenal	1360	1970	Metallic	NB	4	8	8	MS/RI/O/ST
40	Vanillin	1410	2601	Vanilla-like	A	256	256	256	MS/RI/O/ST

*ND* not detected, *CB* control bagel, *BF*_*24*_ 24 h fermented bagel, *BF*_*48*_ 48 h fermented bagel, *NB* neutral basic fraction, *A* acidic fraction

^a^ Compound identified by comparison of its odour quality and intensity, retention indices on capillaries DB-5 and FFAP as well as mass spectra in EI with data of reference compounds. MS, RI, O, ST represents mass spectra, retention indices, olfactometry and standard odorants respectively

^b^ Odour quality as perceived at the sniffing port

### Aroma-active compounds in long, cold fermented bagels

Application of long, cold fermentation (5 °C, 24 h and 48 h) produced bagels that exhibited a wider range of FD factors (4–1024) than the control bagel (Table 2). For instance, the FD factor of 2,3-butanedione in the 24 h and 48 h fermented bagels increased by almost (4 times) and 8 times the value obtained in the control bagel. Other compounds exhibiting higher FD factors in the long, cold fermented bagels were; acetic acid (sweaty), 2/3-methylbutanal (malty), 2,3-butanedione (buttery), propionic acid (sweaty/pungent), butanoic acid (sweaty), acetoin (buttery), 3-methylbutanol (malty), furfural (bread-like), 2-methyl pyrazine (nutty), 2/3-methyl butanoic acid (sweaty), methional (baked potato-like), 2-acetyl-1-pyrroline (roasty), benzaldehyde (almond-like), (*Z*)-4-heptenal (biscuit-like), acetyl-pyrazine (toasty), 4-HDMF (sweet/caramel), benzyl alcohol (sweet/flowery), phenyl acetaldehyde (rose-like), 2-phenyl ethanol (honey-like), octanoic acid (fatty), 4-vinyl-2-methoxyphenol (smoky) and vanillin (vanilla-like) all of which exhibited FD factors from16 to 1024. While long, cold fermented bagels generally exhibited higher FD values than the control, the 48 h bagel also showed higher FD values in some compounds (i.e. diacetyl, 1-butanol, 2/3-methylbutanoic acid, heptanol, (Z)-4-heptenal and 2-pentyl furan) compared to the 24 h bagels.

The influence of fermentation temperatures on the formation of volatile compounds in bread crust and crumb has been well documented [47–49]. While high fermentation temperatures (≥ 27 °C) are more suitable for generating more complete volatile profiles, most bread industries are more favorable to employing longer fermentation time or using sourdough that needs time to ferment. For instance, Zehentbauer and Grosch [48] observed that when bread is prepared from dough subjected to an initial 2 h of fermentation at 22 °C and an additional 18 h of fermentation at 4 °C, the resulting bread exhibited similar amounts of Strecker aldehydes (i.e. 2-methylpropanal, 2-methylbutanal and 3-methylbutanal) as obtained with the artisanal process. This observation is probably due to a longer proteolysis which leads to the formation of amino acids that participates in the Strecker reactions as well as the Ehrlich pathway to produce the aldehydes. It is worthy of note that both 2,3-butanedione and HDMF which exhibited the highest FD factors in the cold fermented bagels as well as many other key aroma compounds such as: 2/3-methylbutanal, acetoin, 3-methylbutanol, furfural, 2-methyl pyrazine, isoamyl acetate, methional, 2-acetyl-1-pyrroline, benzaldehyde, (*Z*)-4-heptenal, acetyl pyrazine, phenyl acetaldehyde and vanillin have been identified in the crumb of wheat bread [3, 11, 47]. Also, various acids such as acetic acid, butanoic acid, 2/3-methyl butanoic acid and octanoic acid which exhibited high FD factors ≥ 16 in the cold fermented bagels have been reported in bread [50, 51].

### Quantitation and odour-activity values (OAVs) of aroma-active compounds in bagels

To have an insight into the contribution of each compounds to the overall aroma of bagels, 22 aroma-active compounds with FD factors ≥ 16 were selected for further investigation. For each of the selected compound, a stable isotopologue (Table 1) was employed as an internal standard to quantify it. As expected the long cold fermented bagels produced compounds with significantly (p < 0.05) high concentrations (Table 3). The highest concentrations (1126–12,950 μg kg^−1^) were determined for 2,3-butanedione, 2-phenylethanol, 3-methylbutanal and acetoin respectively (Table 3). The lowest concentrations (17–43 μg kg^−1^) were obtained for phenyl acetaldehyde, methional and 2-acetyl-1-pyrroline respectively. A comparative analysis of the aroma potencies between the three differently produced bagels revealed some differences. Cold fermented bagels showed more potencies for the buttery smelling 2,3-butanedione, baked potato-like methional and the toasty-like 2-acetyl-1-pyrroline as revealed by their respective high odour-activity values (Table 3). For example, 2-acetyl-1-pyrroline exceeded its threshold by factors of 2603 and 2466 in the 24 h and 48 h cold fermented bagels respectively. 2-Acetyl-1-pyrroline only exceeded its threshold by a factor of 2329 in the control bagels. Similarly, 2,3-butanedione exceeded its threshold by factors of 1815 and 1992 in the 24 h and 48 h cold fermented bagels respectively. On the other hand 2,3-butanedione only exceeded its threshold by a factor of 109 in the control bagel. Similar trend was noticed with the methional, acetyl pyrazine, HDMF, 4-vinyl-2-methoxyphenol, vanillin, 2/3-methylbutanal, 2-phenyl ethanol, butanoic acid, 3-methylbutanol and benzaldehyde. However, acetic acid, phenyl acetaldehyde had OAVs below 1.

**Table 3:** Concentrations, odour thresholds and odour activity values (OAVs) of key aroma compounds (FD factor ≥ 16) in cold fermented bagels

No.	Compounds	Concentration (μg kg^−1^)			Threshold in starch (μg kg^−1^)	Odour activity values (OAVs)^c^		
		CB	BF_24_	BF_48_		CB	BF_24_	BF_48_
1	Acetic acid	300 ± 2.0^c^	480 ± 2.0^b^	510 ± 2.0^a^	31,140	< 1	< 1	< 1
2	2,3-Butanedione (diacetyl)	710 ± 5.1^c^	11,800 ± 12.0^b^	12,950 ± 15.5^a^	6.5	109	1815	1992
3	2/3-Methyl butanal	164 ± 2.1^c^	321 ± 2.1^b^	434 ± 2.0^a^	32^a^	5	10	14
4	Butanoic acid	113 ± 1.0^c^	201 ± 1.0^b^	317 ± 1.0^a^	100^a^	1	2	3
5	Acetoin	1140 ± 4.5^c^	1245 ± 5.0^b^	1276 ± 4.0^a^	Nf	nd	nd	nd
6	3-Methyl butanol	647 ± 3.1^c^	1126 ± 7.8^b^	1364 ± 10.0^a^	102	6	11	13
7	Furfural	101 ± 1.0^b^	126 ± 1.0^a^	124 ± 1.0^a^	Nf	nd	nd	nd
8	2-Methylpyrazine	30 ± 0.2^c^	54 ± 0.2^b^	76 ± 0.2^a^	Nf	nd	nd	nd
9	3-Methylbutanoic acid	64 ± 1.0^c^	276 ± 2.1^b^	314 ± 2.0^a^	24	3	12	13
10	Methional	16 ± 0.1^c^	24 ± 0.1^b^	43 ± 0.1^a^	0.27	59	89	160
11	2-Acetyl-1-pyrroline	17 ± 0.1^ab^	19 ± 0.1^a^	18 ± 0.1^a^	0.0073^a^	2329	2603	2466
12	Benzaldehyde	174 ± 3.0^c^	920 ± 4.5^b^	1121 ± 6.0^a^	350	< 1	3	3
13	5-Methyl-2-furanmethanol	46 ± 0.1^c^	52 ± 0.1^b^	58 ± 0.1^a^	11.9^b^	4	4	5
14	(*Z*)-4-Heptenal	51 ± 0.1^c^	135 ± 0.2^b^	234 ± 0.2^a^	3	17	45	78
15	Acetyl pyrazine	171 ± 1.0^c^	186 ± 1.0^b^	193 ± 1.0^a^	nf	nd	nd	nd
16	4-Hydroxy-2,5-dimethyl-3(2H)-furanone	234 ± 2.0^c^	347 ± 2.1^b^	453 ± 3.0^a^	13 ^b^	18	27	35
17	Benzyl alcohol	115 ± 1.0^c^	176 ± 2.0^b^	182 ± 2.1^a^	nf	nd	nd	nd
18	Phenyl acetaldehyde	15 ± 0.0^b^	18 ± 0.1^a^	17 ± 0.1^a^	28^b^	< 1	< 1	< 1
19	2-Phenyl ethanol	1101 ± 5.0^c^	1134 ± 5.0^b^	1512 ± 5.0^a^	125^b^	9	9	12
20	Octanoic acid	87 ± 2.1^c^	102 ± 2.0^b^	116 ± 2.0^a^	Nf	nd	nd	nd
21	4-Vinyl-2-methoxyphenol	146 ± 2.0^c^	305 ± 2.1^b^	512 ± 4.0^a^	18^b^	8	17	28
22	Vanillin	56 ± 0.1^c^	73 ± 0.1^b^	95 ± 0.1^a^	4.6^b^	12	16	21

*nf* not found, *nd* not determined,* CB* control bagel, *BF*_*24*_ 24 h fermented bagels, *BF*_*48*_ 48 h fermented bagels; Mean ± SD; superscripts with different letters in a row are significantly (p < 0.05) different

^a^Reference; Zehentbauer and Grosch [48]

^b^Reference; Rychlik and Grosch [10]

^c^OAV on the basis of odour thresholds in starch

While some of the bagel aroma compounds were already present in the wheat flour and were thus transferred into the bagel. Others such as 3-methylbutanol, 2-phenyl ethanol and 2,3-butanedione were probably formed during biochemical reactions in the yeast metabolism during the dough fermentation [27]. On the other hand the nitrogen-containing compounds such as the roasty 2-acetyl-1-pyrroline and acetyl pyrazine were formed via the reaction of free amino acids l-ornithine or l-proline with dihydroxyacetone phosphate [52]. In addition to the nitrogen-containing compounds, aldehydes, such as 2/3-methylbutanal (malty), phenyl acetaldehyde (rose/floral) and methional (baked potato-like) were formed by the Strecker degradation of valine, isoleucine, leucine, phenylalanine and methionine respectively [53]. Moreover the caramel-like 4-Hydroxy-2,5-dimethyl-3(2H)-furanone (HDMF) can be formed by the Maillard reaction [54]. 4-Hydroxy-2,5-dimethyl-3(2H)-furanone is mainly formed via Maillard reaction of pentoses with the amino acids glycine and alanine, respectively. Alternatively, 4-hydroxy-2,5-dimethyl-3(2H)-furanone can also be produced without the direct interaction of glycine [36]. Furthermore, certain aldehydes such as (*E,E*)-2,4-decadienal, (*E*)-2-nonenal, and (*E*)-4,5-epoxy-(*E*)-2-decenal were formed by autoxidation and thermal degradation of fatty acids respectively [53].

### Sensory analysis and aroma reconstitution evaluation

**Fig. 3:**
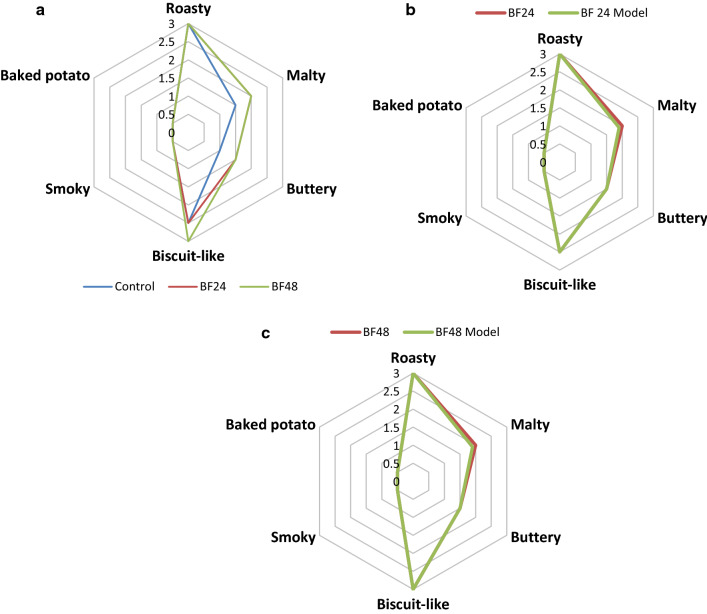
**a** Aroma profiles of bagels; control bagels (blue line), 24 h fermented bagels (red line) and 48 h fermented bagels (green line). **b** A comparative aroma profiles of 24 h bagels (red colour) and its aroma model (green colour). **c** Aroma profiles of 48 h bagel (red colour) and its aroma model (green colour)

The results of sensory evaluation of the different bagels (i.e. control, 24 h fermented and 48 h fermented) are shown in (Fig. 3a, Table 4). In order to select the final descriptors, all the panelists had to achieve complete agreement on any given descriptor for such descriptor to be chosen. The aroma profiles of the cold fermented bagels were characterized as roasty, biscuit-like, malty, smoky and buttery. The control bagel exhibited similar but less intense aroma notes as compared to the cold fermented bagels. However, the 24 h and 48 h bagels flavor profiles were similar with the exception of the biscuit-like aroma note (Table 4). The statistical analysis results (Table 4) showed that the six attributes (roasty, malty, buttery, biscuit-like, smoky and baked potato like) with different superscripts provided a clearer explanation of the aroma characteristics of the different bagels. To confirm this observation, recombination experiments were carried out by mixing solutions of the pure reference compounds in the same amounts as indicated for both 24 h and 48 h bagels respectively (Table 5). A parallel evaluation of the recombination models of the freshly baked 24 h and 48 h bagels was conducted. Results showed that the recombinant model imitated well the flavor of the freshly baked bagels (Fig. 3b, c, Table 4). The aroma of the recombination models had good similarities for all the odor notes such as roasty, baked potato-like, smoky and biscuit-like. The roasty and biscuit-like aroma notes were perceived as equally intense in the aroma models as well as in the bagels.

**Table 4:** The mean scores of the six attributes for the three bagels and the aroma models generated. (Supplementary)

Sensory attribute	Bagels			Mean scores of bagel’s and their aroma models			
	Control	BF_24_	BF_48_	BF_24_	BF_24_ Model	BF_48_	BF_48_ Model
Roasty	3.0 ± 0.21^A^	3.0 ± 0.70^A^	3.0 ± 0.91^A^	3.0 ± 0.42^a^	3.0 ± 0.50^a^	3.0 ± 0.72^a^	3.0 ± 0.23^a^
Malty	1.5 ± 0.05^B^	2.0 ± 0.23^A^	2.0 ± 0.60^A^	2.0 ± 0.23^a^	1.9 ± 0.14^a^	2.0 ± 0.33^a^	1.9 ± 0.24^a^
Buttery	1.0 ± 0.04^B^	1.5 ± 0.02^A^	1.5 ± 0.13^A^	1.5 ± 0.05^a^	1.5 ± 0.25^a^	1.5 ± 0.15^a^	1.5 ± 0.21^a^
Biscuit-like	2.5 ± 0.81^B^	2.5 ± 0.33^B^	3.0 ± 0.56^A^	2.5 ± 0.50^a^	2.5 ± 0.71^a^	3.0 ± 0.30^a^	3.0 ± 0.80^a^
Smoky	0.5 ± 0.02^A^	0.5 ± 0.01^A^	0.5 ± 0.03^A^	0.5 ± 0.04^a^	0.5 ± 0.12^a^	0.5 ± 0.03^a^	0.5 ± 0.05^a^
Baked potato	0.5 ± 0.01^A^	0.5 ± 0.03^A^	0.5 ± 0.01^A^	0.5 ± 0.02^a^	0.5 ± 0.04^a^	0.5 ± 0.01^a^	0.5 ± 0.03^a^

^A, B, C^^: a^^, b, c^Different letters within the same row represents significant differences (p < 0.05) using Duncan’s multiple comparison test (n = 30, 10 panellists with 3 replications)

*BF*_*24*_ 24 h fermented bagel, *BF*_*48*_ 48 h fermented bagel

**Table 5:** Aroma models composition for bagels produced from 24 and 48 h cold fermentation

No.	Compounds	Concentration^b^ (μg kg^−1^)	
		BF_24_	BF_48_
1	Acetic acid	480	510
2	2,3-Butanedione (diacetyl)	11,800	12,950
3	2/3-Methyl butanal	321	434
4	Butanoic acid	201	317
5	Acetoin^a^	1245	1276
6	3-Methyl butanol	1126	1364
7	3-Methylbutanoic acid	276	314
8	Methional	24	43
9	2-Acetyl-1-pyrroline	19	18
10	Benzaldehyde	920	1121
11	5-Methyl-2-furanmethanol	52	58
12	(*Z*)-4-Heptenal	135	234
13	4-Hydroxy-2,5-dimethyl-3(2H)-furanone	347	453
14	Phenyl acetaldehyde	18	17
15	2-Phenyl ethanol	1134	1512
16	4-Vinyl-2-methoxyphenol	305	512
17	Vanillin	73	95

^a^Acetoin was included in the model even though its threshold in starch was not found

^b^Ethanolic solutions of aroma compounds dissolved in free corn starch

### Omission tests

The contributions of some key aroma compounds to the flavor of the bagels, was evaluated by omission tests. Omission tests are used to assess the contribution of individual compound to the overall aroma of a given food [54]. Eleven aroma omission models (M1–M11), containing either single or a group of compounds, were prepared. Each of the omission models was analyzed in triangular experiments with two complete recombination models (Table 6). Results showed that, the omission of the entire group of acids (M1) from the complete recombination model could be distinguished by 9 out of the 10 assessors. This shows that these acids (i.e. acetic acid, butanoic acid and 3-methyl butanoic acid) play an important role in the overall aroma of the long, cold fermented bagels. In the second group, the ketones (2,3-butanedione and acetoin) with characteristic buttery nuance were omitted. Acetoin was included in this group because of its high concentration. Result of the omission of the entire ketones from the complete recombination model showed that all 10 assessors could detect between the omission model and the complete recombination models. This shows that 2,3-butanedione and acetoin greatly influence the overall aroma of the bagel. When the aldehydes (M3) (2,3-methyl butanal, methional, benzaldehyde, (Z)-4-heptenal, phenyl acetaldehyde and vanillin) were omitted, only 8 assessors were able to detect the difference (p < 0.01). Similar trend was observed when the entire group of alcohols (M4) was omitted. In model 5, 4-vinyl-2-methoxyphenol was omitted because of its high concentration and the result showed that only 7 assessors were able to detect the difference between the omission model and the complete recombination models. In model 6, 4-hydroxy-2,5-dimethyl-3(2H)-furanone was omitted and this resulted in significant (p ≤ 0.001) reduction in the characteristic aroma of the bagels. In addition, 9 of the assessors were able to distinguish its omission from the complete recombination models. Similar observation was obtained when other single compounds such as 2-phenyl ethanol, methional, (Z)-4-heptenal, 5-methyl-2-furanmethanol and 2-acetyl-1-pyrroline were omitted from the complete recombination models respectively. However, the omission of 5-methyl-2-furanmethanol and 2-acetyl-1-pyrroline was detected by all 10 assessors.

**Table 6:** Omission analysis on the bagel aroma models (BF_24_ and BF_48_)

Odorant groups	Aroma note	Compounds omitted	No of correct judgments^a^ BF_24_	No of correct judgments^a^ BF_48_	Significance^b^
Acids (M1)	Sweaty	Acetic acid, butanoic acid, 3-methylbutanoic acid	9/10	9/10	***
Ketones (M2)	Buttery	2,3-Butanedione, acetoin	10/10	10/10	***
Acetaldehydes (M3)	Malty, baked potato, almond-like, biscuit-like, vanilla	2,3-Methylbutanal, methional, benzaldehyde, (Z)-4-heptenal, phenyl acetaldehyde, vanillin	8/10	8/10	**
Alcohols (M4)	Malty, bread-like, honey	3-Methylbutanol, 5-methyl-2-furanmethanol, 2-phenyl ethanol	8/10	8/10	**
Phenol (M5)	Smoky	4-Vinyl-2-methoxyphenol	7/10	7/10	*
(M6)	Sweat, caramel	4-Hydroxy-2,5-dimethyl-3(2H)-furanone	9/10	9/10	***
(M7)	Floral, honey	2-Phenyl ethanol	8/10	8/10	**
(M8)	Cooked potato-like	Methional	8/10	8/10	**
(M9)	Biscuit-like	(Z)-4-Heptenal	9/10	9/10	***
(M10)	Bread-like	5-Methyl-2-furanmethanol	10/10	10/10	***
(M11)	Popcorn-like	2-Acetyl-1-pyrroline	10/10	10/10	***

*M1–M11* Models

^a^Number of correct judgments from 10 assessors

^b^Significance: * significant (α ≤ 0.05); **, highly significant (α ≤ 0.01); ***, very highly significant (α ≤ 0.001)

## Conclusion

This study has revealed the key aroma-active compounds responsible for the characteristic aroma of the long, cold fermented bagels. The results of the OAVs and sensory studies showed distinct differences in the aroma notes of the cold fermented and control bagels. Whilst the cold fermented bagels exhibited roasty, malty, buttery, baked potato-like, smoky and biscuit-like notes, the odour notes in the control bagels were similar to the other bagels but less intense. Aroma compounds such as 2,3-butanedione (buttery), acetoin (buttery), 2-acetyl-1-pyrroline (roasty), 5-methyl-2-furanmethanol (bread-like), (Z)-4-heptenal (biscuit-like) and HDMF, were the key aroma compounds. In addition, vanillin (vanilla), 2/3-methylbutanal (malty), 3-methyl butanoic acid (sweaty), 3-methylbutanol (malty), methional (baked potato-like), 2-phenyl ethanol (honey-like), benzaldehyde (almond-like), and butanoic acid (sweaty) were identified as important aroma compounds of bagels. These finding establishes a basis for further research on the effect of cold fermentation on bakery products found in many world cuisines.

## Data Availability

All data generated or analyzed during this study are included in this published article.
